# Transcriptome Sequencing and Analysis for Culm Elongation of the World’s Largest Bamboo (*Dendrocalamus sinicus*)

**DOI:** 10.1371/journal.pone.0157362

**Published:** 2016-06-15

**Authors:** Kai Cui, Haiying Wang, Shengxi Liao, Qi Tang, Li Li, Yongzhong Cui, Yuan He

**Affiliations:** 1 Research Institute of Resources Insects, Chinese Academy of Forestry, Kunming, 650224, People’s Republic of China; 2 Hunan Co-Innovation Center for Utilization of Botanical Functional Ingredients, Hunan Agricultral University, Changsha, 410128, People’s Republic of China; Institute of Crop Sciences, CHINA

## Abstract

*Dendrocalamus sinicus* is the world’s largest bamboo species with strong woody culms, and known for its fast-growing culms. As an economic bamboo species, it was popularized for multi-functional applications including furniture, construction, and industrial paper pulp. To comprehensively elucidate the molecular processes involved in its culm elongation, Illumina paired-end sequencing was conducted. About 65.08 million high-quality reads were produced, and assembled into 81,744 unigenes with an average length of 723 bp. A total of 64,338 (79%) unigenes were annotated for their functions, of which, 56,587 were annotated in the NCBI non-redundant protein database and 35,262 were annotated in the Swiss-Prot database. Also, 42,508 and 21,009 annotated unigenes were allocated to gene ontology (GO) categories and clusters of orthologous groups (COG), respectively. By searching against the Kyoto Encyclopedia of Genes and Genomes Pathway database (KEGG), 33,920 unigenes were assigned to 128 KEGG pathways. Meanwhile, 8,553 simple sequence repeats (SSRs) and 81,534 single-nucleotide polymorphism (SNPs) were identified, respectively. Additionally, 388 transcripts encoding lignin biosynthesis were detected, among which, 27 transcripts encoding Shikimate O-hydroxycinnamoyltransferase (HCT) specifically expressed in *D*. *sinicus* when compared to other bamboo species and rice. The phylogenetic relationship between *D*. *sinicus* and other plants was analyzed, suggesting functional diversity of HCT unigenes in *D*. *sinicus*. We conjectured that HCT might lead to the high lignin content and giant culm. Given that the leaves are not yet formed and culm is covered with sheaths during culm elongation, the existence of photosynthesis of bamboo culm is usually neglected. Surprisedly, 109 transcripts encoding photosynthesis were identified, including photosystem I and II, cytochrome b6/f complex, photosynthetic electron transport and F-type ATPase, and 24 transcripts were characterized as antenna proteins that regarded as the main tool for capturing light of plants, implying stem photosynthesis plays a key role during culm elongation due to the unavailability of its leaf. By real-time quantitative PCR, the expression level of 6 unigenes was detected. The results showed the expression level of all genes accorded with the transcriptome data, which confirm the reliability of the transcriptome data. As we know, this is the first study underline the *D*. *sinicus* transcriptome, which will deepen the understanding of the molecular mechanisms of culm development. The results may help variety improvement and resource utilization of bamboos.

## Introduction

Currently, due to depleting fossil reserves and increasing emission of greenhouse gases, it is obvious that utilization of renewable feedstock is one necessary step towards a sustainable development of our future [1]. Therefore, the efforts to exploit a variety of potential plant feedstocks for the energy, chemical, and food ingredients have been made particularly from agricultural and forestry biomass resources [2,3,4]. *Dendrocalamus sinicus*, belonging to Bambusoideae of Gramineae, is the world’s largest bamboo species with strong woody stems (maximal diameter 30 cm and maximal height 33 m), which is mainly distributed in the southwest region of China [5]. It is called Da bamboo by local residents, and its culms yield per unit area is 5–8 times as that of *Phyllostachys pubscens* that is now popularized as a major economic bamboo species in China [6]. In its native area, *D*. *sinicus* is economically important as a raw material for furniture, construction, and industrial paper pulp [7]. Because of easy propagation, fast growth, and high productivity, it is considered as one of the most potential renewable non-woody lignocellulosic feedstocks for bioenergy and biorefinery [8].

The culm growth of *D*. *sinicus* is extremely amazing: shooting initiate from early June, and height growth of culm end in late August. By the brief three months, new shoots can reach the height and diameter of adult mother bamboo—near to 30 cm of diameter and 30 m of height. To our knowledge, no any plant in this planet has faster growth rate compared with *D*. *sinicus*.

In terms of the bamboo growth, although some molecular researches were reported, such as the protein expression profiles [9], cloning of a set of cDNA sequences [10,11,12,13,14,15,16], Expressed Sequence Tags (EST) [17], RNA-seq [18,19], monoclonal antibody banks [20] and draft genome [21], the molecular mechanism related to culm development is still unclear. Especially, to date, there was almost no molecular data of *D*. *sinicus* except SSR marker development [22], which seriously restricted its variety improvement and resource development.

Transcriptomic analyses are extremely efficient methods for identifying differential expression genes at the whole-genome level. In contrast to the traditional fragment analysis techniques, RNAs-seq have some advantages: (1) high efficient and low cost; (2) cataloguing all kinds of transcripts including mRNAs, noncoding RNAs, and small RNAs; (3) investigating the transcriptional structure of genes, splicing patterns, and gene isoforms; (4) studying posttranscriptional modification and mutations; and (5) precisely quantifying gene expression in large-scale at the same time of sequencing. Moreover, RNA-seq is genome-independent and is especially useful for analyzing the transcriptome of a species without complete genome information [23,24].

In this study, the high throughput RNA-Seq was performed to elucidate the molecular processes involved in the rapid culm elongation of *D*. *sinicus* during an integrated growing season. Also, based on the transcriptome analysis, a series of results were displayed including novo assembly, gene characterization, gene classification, gene enrichment, SSR and SNP marker identification. Given the unique growth characteristics of *D*. *sinicus*, this study will lay foundation for deep understanding of bamboo growth.

## Materials and Methods

### Plant materials

Culm samples were collected from natural population of *D*. *sinicus* located at Ninger county in Yunnan province of China (23°07′~23°09′ N, 101°04′~101°08′ E), where the *D*. *sinicus* distributed typically. The sampling site belongs to public forest, and this field sampling was permitted by Ninger County Government. Based on the depiction by Banik (2015) [25], the internodal elongation begins at the basal portion of the culm and then gradually proceeds to the top, that is, elongation is mainly due to the intercalary meristem present at the node. Development, maturation, and aging gradually completed from basal to top internodes. Thus, the basal internode represents the higher lignified degree compared with middle and top internodes. In intercalary growth, the immature axis increases in length by the elongation of cells in zones of secondary meristems each located just above the node. Thus, different internode represents different developmental condition. Therefore, basal culms from different internode originated from a same bamboo were harvested. A total of nine internodes were selected and triplicate replicate was mixed. Samples were washed with deionized water, and wiped with filter paper, then, immediately frozen in liquid nitrogen and stored at −80°C until analysis.

### RNA extraction, library construction and RNA-seq

Total RNA of each sample was isolated separately using Trizol Reagent (Invitrogen, Carlsbad, CA, USA) following the manufacturer’s protocol. The purified RNA concentration was quantified by a spectrophotometer (UV-Vis Spectrophotometer, Quawell Q5000, San Jose, CA, USA), and the purity and degradation of total RNA were checked on 1% agarose gels before proceeding. For maximizing the diversity of transcriptional units, RNA from each sample was mixed into a single pool. The mRNA-seq library was constructed using Illumina’s TruSeq RNA Sample Preparation Kit (Illumina Inc, San Diego, CA, USA). Shortly, mRNA was purified by oligo (dT) magnetic beads. After purification, the mRNA was fragmented into small pieces using divalent cations under elevated temperature and the cleaved RNA fragments were used for first strand cDNA synthesis using reverse transcriptase and random primers. This was followed by second strand cDNA synthesis using DNA polymerase I and RNaseH. Then, cDNA fragments were perfored an end repair process and ligation of adapters. The product was purified and enriched with PCR to create the final cDNA library [26]. Finally, the cDNA library was sequenced by the Illumina HiSeq^™^ 2000 sequencing platform.

### Analysis of Illumina sequencing results

The raw reads from mRNA-seq were filtered by discarding the reads with adaptor contamination, masking low-quality reads with ambiguous ‘N’ bases and removing the reads in which more than 10% bases had a Q-value <20 [27]. The clean reads were assembled into contigs using the Trinity program [28]. In the Trinity method, an optimized k-mer length of 25 was used for de novo assembly. According to the paired-end information of the sequences, the contigs were linked into transcripts. Subsequently, the transcripts were clustered based on nucleotide sequence identity, and the longest transcripts in the cluster units were regarded as unigenes to eliminate redundant sequences.

### Functional annotation

A BLASTx search (E≤10^−5^) with unigenes was performed against protein databases such as non-redundant protein (Nr), SwissProt, KEGG and Cluster of Orthologous Groups of proteins (COG), and the best aligning results used to determine sequence direction of unigenes. ESTScan was used to predict its coding regions and sequence direction if a unigene was not aligned in any of the above databases [29]. To reflect the molar concentration of a transcript by normalizing for RNA length and for the total read number, the expression abundance of the unigenes was represented using the number of fragments per kilobase of transcript per million fragments mapped (FPKM) [30]. Functional annotation by gene ontology terms (GO, http://www.geneontology.org) was analyzed by Blast2GO [31] and WEGO [32] software. The KEGG pathways annotation was performed using Blastall software against the KEGG database. KEGG pathways were retrieved from KEGG web server (http://www.genome.jp/kegg/).

### Detection of SSR and SNP markers

To dig the potential SSR markers, the assembled sequences were searched using MISA software (http://pgrc.ipk-gatersleben.de/misa/). The parameters were designed for the identification of perfect dinucleotide motifs with a minimum of six repeats, and tri-, tetra-, penta-, and hexanucleotide motifs with a minimum of five repeats [33,34]. Also, SOAPsnp was used to detect the single-nucleotide polymorphism (SNP) in the transcript. The program can assemble consensus sequence for the genome of a newly sequenced individual based on the alignment of the raw sequencing reads on the Unigenes. The SNPs can then be identified on the consensus sequence through the comparison with the Unigenes [35].

### Gene validation and expression analysis

To validate the transcript sequence and analyze the temporal expression, six genes were mined from the *D*. *sinicus* transcriptome database including *PsbA*, *PsaB*, *PetB*, *PetF*, *Lhca2* and *F5H*. Of which, the first five genes were involved in photosynthesis and the last one gene was associated with lignin biosynthesis. The expression level of genes in different internodes was detected by Real-time qPCR, and *UBQ* (Ubiquitin) gene used to as the internal control. The mRNA was reverse transcribed into complementary DNA (cDNA) according to the instruction of the Superscript III First-Strand Synthesis System (Invitrogen, Carlsbad, USA). The primers used for qPCR were designed using primer premier 3.0 (http://bioinfo.ut.ee/primer3-0.4.0/) and synthesized from Sangon Biotech Company (Shanghai, China). The corresponding gene names, sequences and primers used for RT-qPCR analysis were displayed in File S1. The qPCR reaction was implemented according to the protocol of SYBR^®^ Premix DimerEraser^™^ (TaKaRa, Dalian, China) by 7300 Real Time PCR System (Applied Biosystems, CA, USA). The cycling conditions of PCR reaction were recommended by the manufacturer (30 s at 95°C, 40 cycles of 95°C for 5 s, and 60°C for 31 s). Three biological replicates of each sample and triplicates of each reaction were performed.

### Alignment and phylogenetic tree building

Hidden Markov Model (HMM) was performed to identify the Shikimate O-hydroxycinnamoyltransferase (HCT) genes of the *D*. *sinicus* transcriptome. The profile of the HCT condensation domain (cl19241) used for the HMM search (HMMER 3.1, http://hmmer.janelia.org/) was downloaded from the Pfam database (http://pfam.sanger.ac.uk/).

A total of 27 HCT sequences were got with an E-value threshold of 0.1. The phylogenetic tree was constructed by neighbor-joining method with 1000 bootstrap trials in MEGA5.0 software.

## Results

### Transcriptome sequencing and de novo assembly

**Fig 1:**
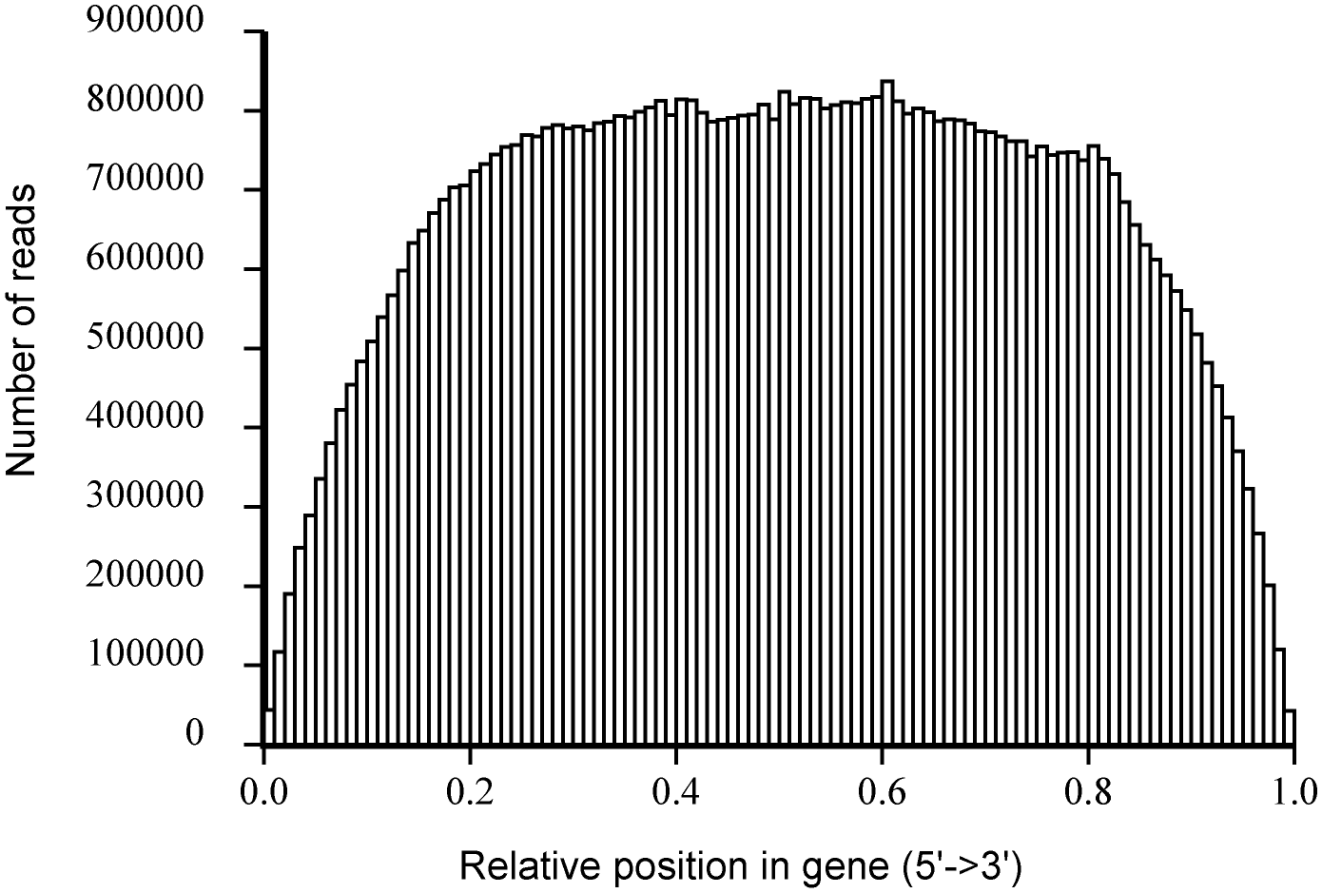
The distribution of the number of reads based on the relative position in gene (5′-3′).

**Fig 2:**
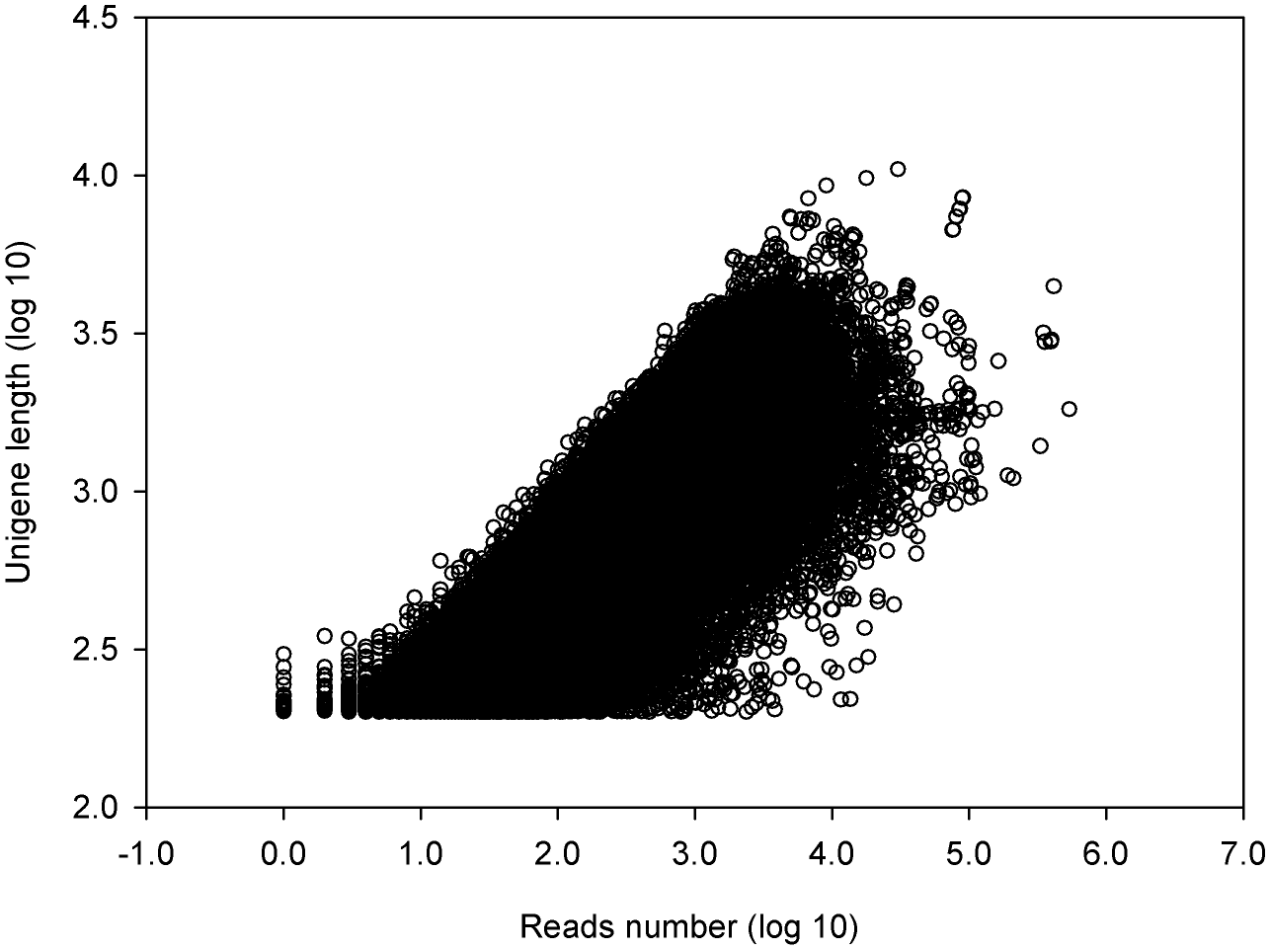
The dependence of unigene lengths on the number of reads assembled into each unigene.

For getting a broad gene library related to culm development, RNA of nine developmental stages of culms was pooled. A total of 65.08 million raw reads and 4.59 gigabase pairs (Gbp) with an average GC content of 53.21% were obtained by a stringent quality check. The reads with Q ≥ 20 and no ambiguous “N” were defined as high-quality reads. Using SOAPdenovo [36], 54,807,622 high-quality reads were assembled into 240,630 contigs after removal of adaptor sequences and exclusion of contaminated or short reads. By the Trinity de novo assembly program, short-read sequences were assembled into 81,744 unigenes with a mean length of 723 bp, of which, 63,130 unigenes (77.23%) with length 200–1000 bp, 14,161 unigenes (17.32%) with length 1000–2000 bp, and 4453 unigenes (5.45%) with length > 2000 bp (Table 1). The N50 values of contigs and unigenes were 310 bp and 1095 bp, respectively. The number of reads based on the relative position in gene (5′-3′) presented normal distribution (Fig 1). There was a positive relationship between the length of a given unigene and the number of reads (Fig 2), indicating a randomly fragmented transcriptome in this study. What’s more, the raw paired-end sequence data with FASTQ format was deposited in the NCBI Sequence Read Archive (SRA) database with accession number SRA302259, facilitating the access and use of the *D*. *sinicus* transcriptome sequencing data.

**Table 1:** Length distribution of contigs, scaffolds and unigenes.

Nucleotides length (bp)	Contigs	Unigenes
0–200	170,136	0
200–300	28,593	21,969
300–400	14,630	14,537
400–500	6,951	7,786
500–600	4,047	5,326
600–700	2,822	4,244
700–800	2,135	3,549
800–900	1,758	3,059
900–1000	1,392	2,660
1000–1500	4,239	9,087
1500–2000	2,033	5,074
2000–2500	975	2,356
2500–3000	443	1,093
> = 3000	476	1,004
Total number	240,630	81,744
Total length (bp)	59,576,539	59,137,193
N50 length (bp)	310	1,095
Mean length (bp)	248	723

### Functional annotation and classification

**Fig 3:**
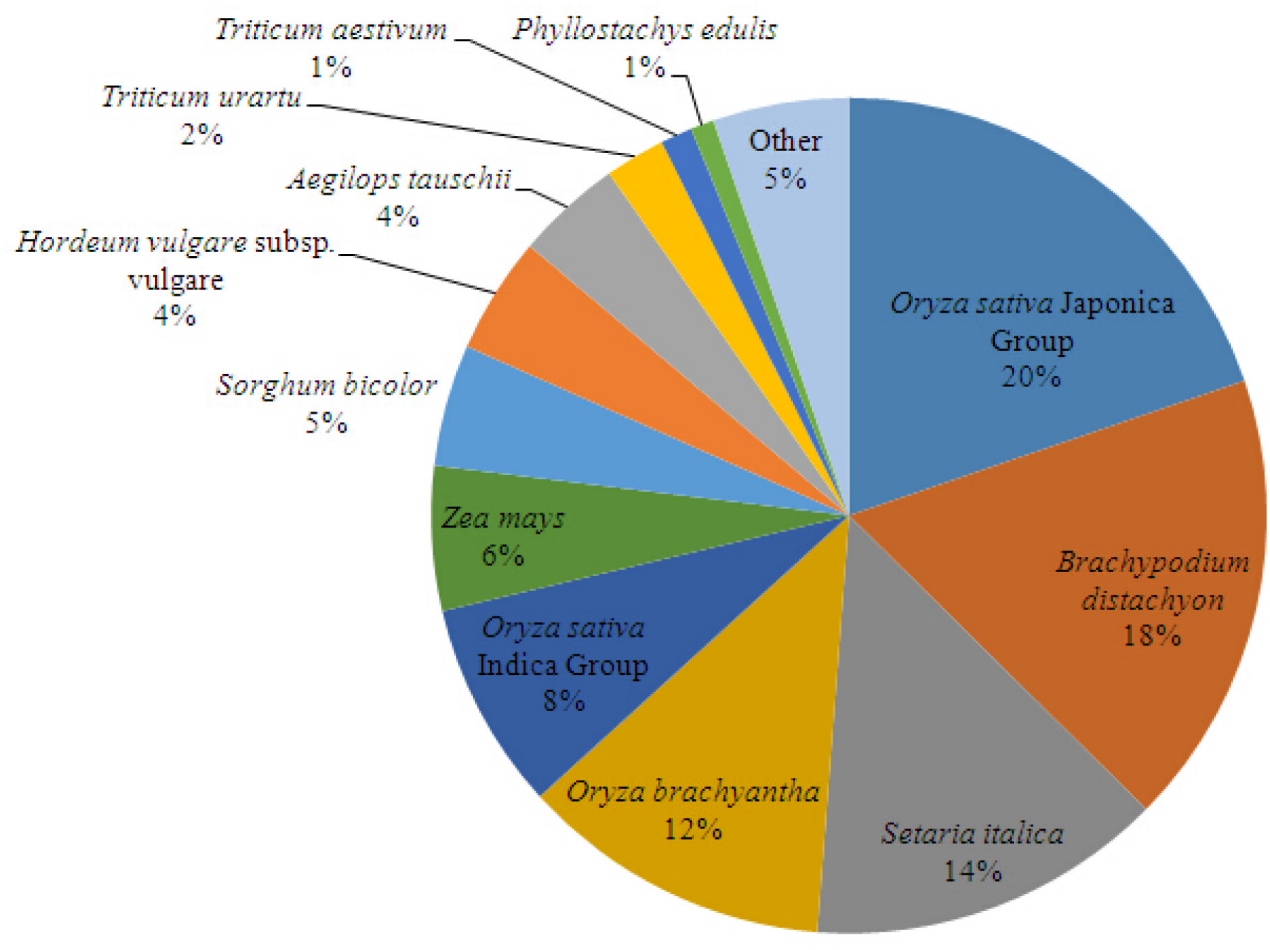
Species distribution of the BLAST hits in Nr dababase. 56,587 BLASTX-hit unigenes were calculated.

A threshold of 10^−5^ was adopted by performing a BLASTX search against diverse protein databases, including the NCBI nonredundant protein (Nr) database, NCBI non-redundant nucleotide sequence (Nt) database, UniProt/Swiss-Prot, Kyoto Encyclopedia of Genes and Genomes (KEGG), Cluster of Orthologous Groups of proteins (COG) and Gene Ontology (GO), and 78.71% of unigenes (64,338) were annotated. According to the BLASTX results, 56,587 (69.22%) unigenes showed significant similarity to Nr protein database, and 61,256 (74.94%) unigenes had homologous proteins in the Nt database. Furthermore, 35,262 (43.14%) unigenes matched the proteins in the Swiss-Prot database (Table 2). Total of 22,569 (40%) unigenes displayed significant homology with sequences of *Oryza*, and 18% and 14% of the mapped sequences have a high similarity with sequences of *Brachypodium distachyon* and *Setaria italica*, respectively. Interestingly, only 509 unigenes (1%) have a high similarity with sequences of *P*. *edulis* (Fig 3).

**Table 2:** Functional annotation of the *D*. *sinicus* transcriptome.

Annotated databases	Unigenes	Percentage of unigenes
nr_Annotaion	56,587	69.22%
nt_Annotaion	61,256	74.94%
swissprot_Annotaion	35,262	43.14%
kegg_Annotaion	33,920	41.50%
COG_Annotaion	21,009	25.70%
GO_Annotaion	42,508	52.00%
Total	64,338	78.71%

**Fig 4:**
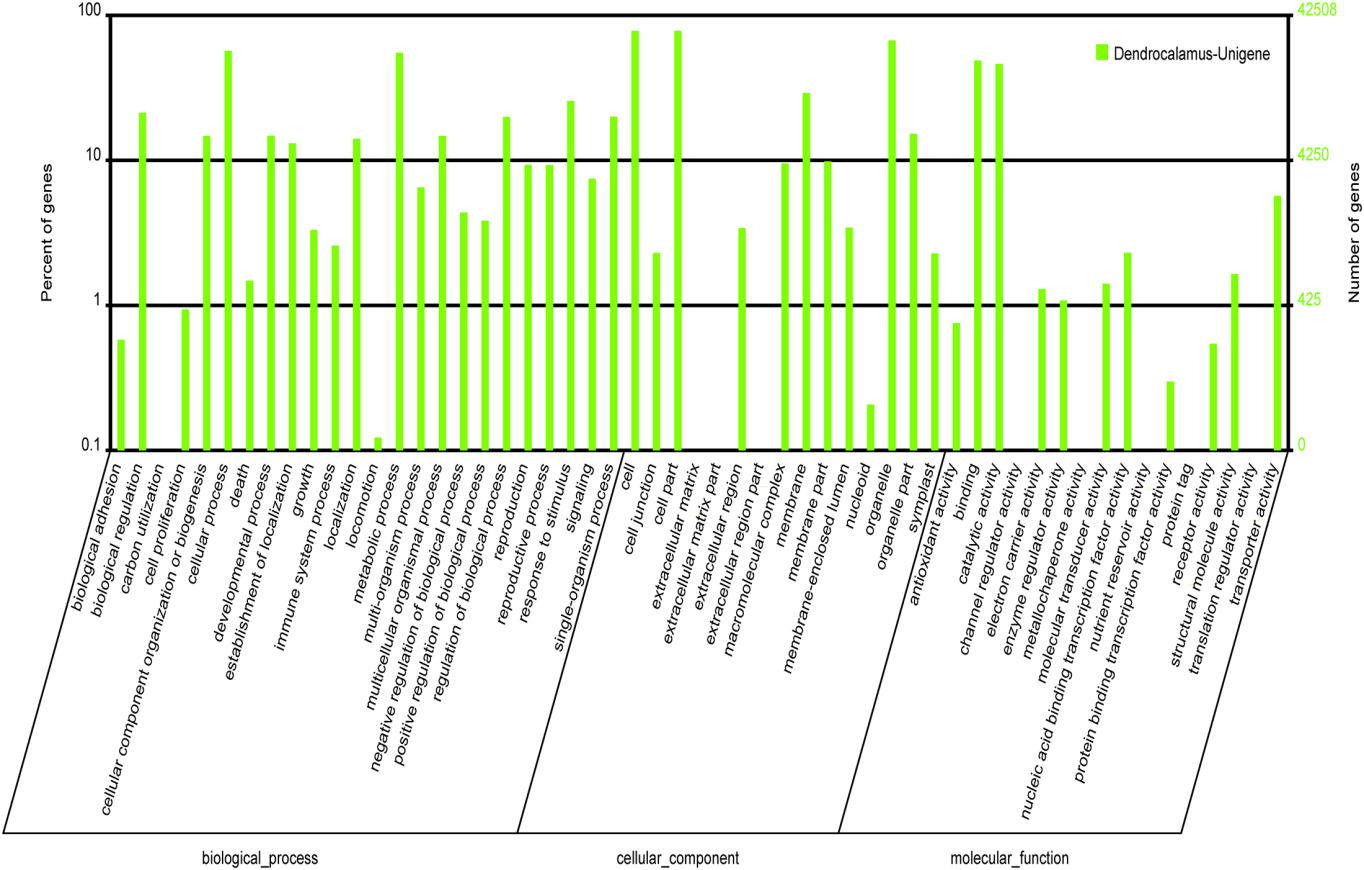
Functional annotation of assembled sequences based on gene ontology (GO) categorization. The unigenes are summarized into three main categories: cellular component, molecular function and biological process.

Overall, GO was used to classify the functions of the assembled transcripts and describe gene products in terms of their associated biological processes, cellular components, and molecular functions [37]. According to GO classification, 42,508 unigenes were divided into the three categories including 52.57% in biological processes, 28.81% in cellular components, and 18.62% in molecular functions (Fig 4). The four largest biological processes were cellular processes, metabolic processes, response to stimulus, and biological regulation. Under the cellular components, the major classifications were cell, cell part, organelle, and membrane. The most of the genes were classified into the molecular functions of binding, catalytic activity, transporter activity, and nucleic acid binding transcription factor activity. These results indicated that culm development was involved in many fundamentally biological regulation and metabolism.

**Fig 5:**
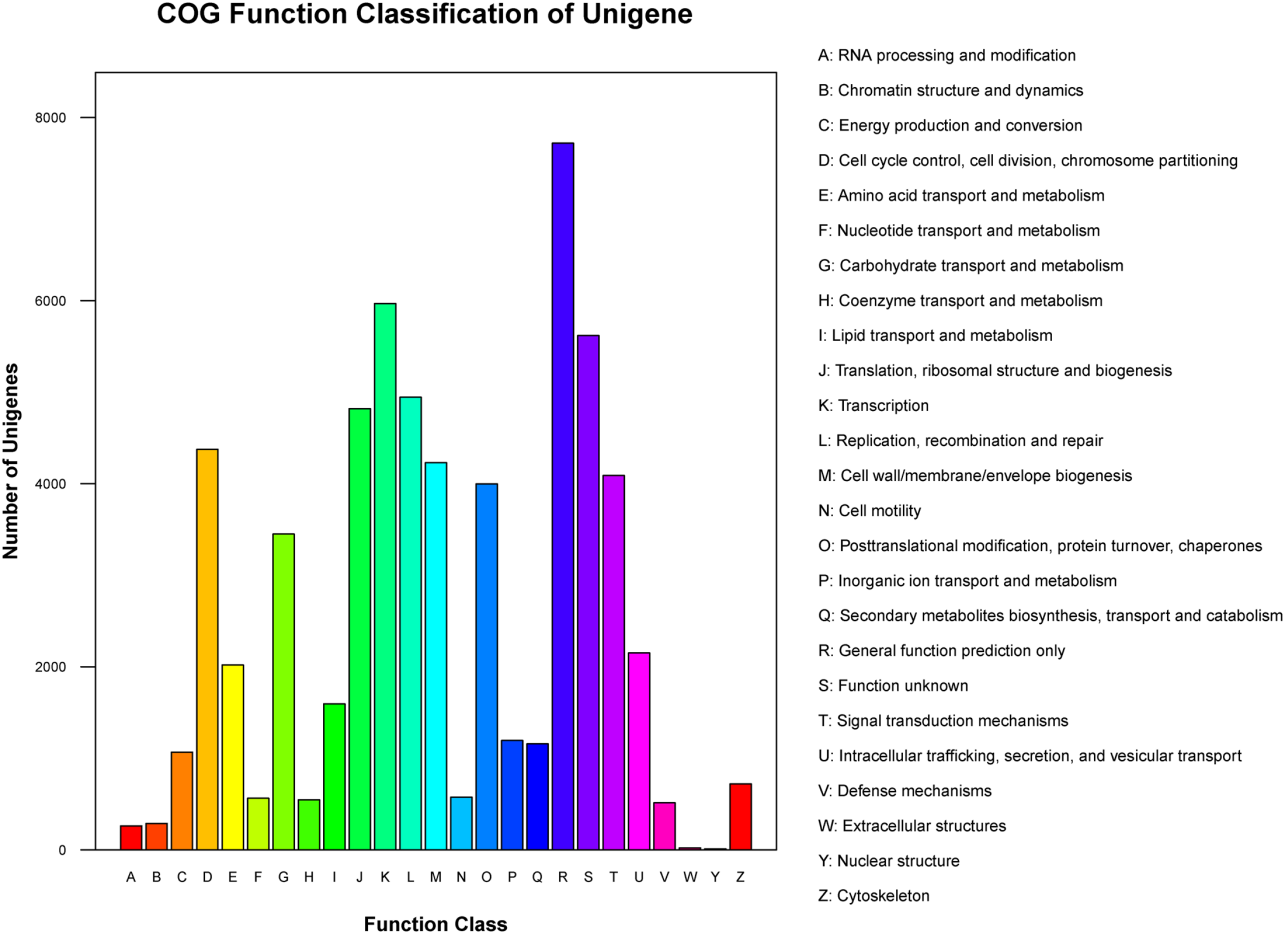
Clusters of orthologous groups (COG) classification. In total, 21,009 of the 81,744 sequences with Nr hits were grouped into 25 classifications.

To predict and classify possible functions, 21,009 of 81,744 (25.70%) unigenes were assigned to the COG database based on Nr annotation, and 25 different functional classes were formed (Fig 5). Of them, the largest group is the cluster for general function prediction (7,720, 36.75%), followed by transcription (5,968, 28.41%), replication, recombination and repair (4,945, 23.54%), translation, ribosomal structure and biogenesis (4,820, 22.94%), cell cycle control, cell division, chromosome partitioning (4,375, 20.82%), cell wall/membrane/envelope/ biogenesis (4,230; 20.13%), and signal transduction mechanisms (4,090, 19.47%), etc. Furthermore, 3450 (16.42%) unigenes were assigned to carbohydrate transport and metabolism. Nevertheless, only 22 and 11 unigenes were assigned to extracellular structures and nuclear structure, respectively.

By KEGG database, gene functions and interactions focused on biochemical pathways can be more easily identified and categorized. A BLASTx with *E* < 10^−5^ was performed against the KEGG database, 33,920 unigenes (41.5%) had significant matches and were assigned to 128 KEGG pathways (S1 Table). In the all categories, the pathways with highest unigene representation were Metabolic pathways (ko01100, 9375 unigenes, 27.64%), followed by RNA transport (ko03013, 4041 unigenes, 11.91%), mRNA surveillance pathway (ko03015, 3424 unigenes, 10.09%) and Endocytosis (ko04144, 3227 unigenes, 9.51%).

### SSR and SNP marker detection

**Fig 6:**
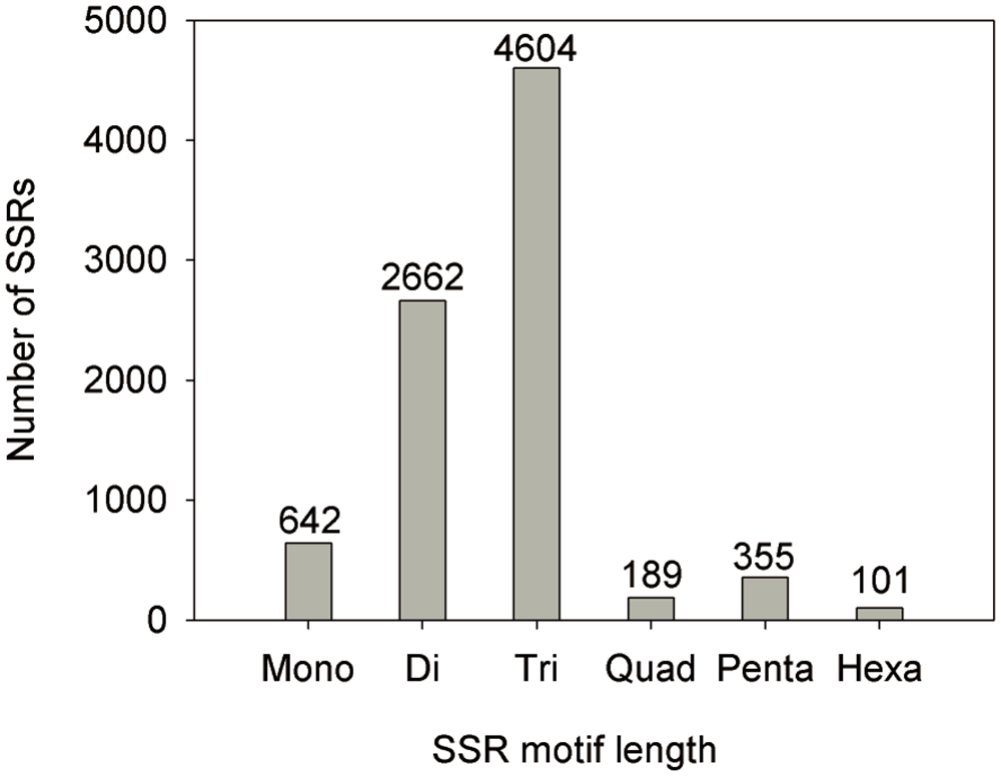
The distribution of SSR motif length.

A total of 7,353 sequences containing 8,553 SSRs were identified from 81,744 unigenes, with 997 unigene sequences containing more than one SSR, and 487 unigene sequences containing SSR present in compound formation. Among them, the number of trinucleotide motifs and dinucleotide motifs were the maximum, accounting for 53.83 and 31.12%, respectively (Table 3). In addition, the most abundant repeat type was AG/CT (1,941), followed by CCG/CGG (1,669), AGG/CCT (918), AGC/CTG (748), GA (611), respectively. However, in the all unigene sequences, the number of quad-, penta- and hexa-nucleotide motifs was less than 5% (Fig 6).

**Table 3:** Frequency of SSRs.

Motif length	Repeat numbers								Total	%
	4	5	6	7	8	9	10	>10		
Mono	0	0	0	0	0	0	0	642	642	7.51
Di	0	0	887	503	432	360	336	144	2662	31.12
Tri	0	3083	1134	338	42	0	2	5	4604	53.83
Quad	0	153	31	0	1	1	3	0	189	2.21
Penta	306	43	6	0	0	0	0	0	355	4.15
Hexa	96	3	1	0	0	0	0	1	101	1.18
**Total**	402	3282	2059	841	475	361	341	792	8553	
**%**	4.70	38.37	24.07	9.83	5.55	4.22	3.99	9.26		

By detection of 81,744 Unigenes (total length 59,137,193 bp) sequence information, a total of 81,534 SNPs were identified with the frequency of 1/725, including transition 52,462 and transversion 29,072. A/G and C/T are the main types, accounting for 32.43% and 31.91% of all the SNPs, respectively. Other four kinds of nucleotide variations (A/C, A/T, C/G and G/T) accounting for 8.89%, 8.03%, 9.85% and 8.88%, respectively (Table 4).

**Table 4:** Statistics of SNP types.

Transition	Number	Transversion	Number
A-G	26,443	A-C	7,250
C-T	26,019	A-T	6,551
		C-G	8,031
		G-T	7,240
Total	52,462		29,072

### Functional genes involved in lignin biosynthesis

**Fig 7:**
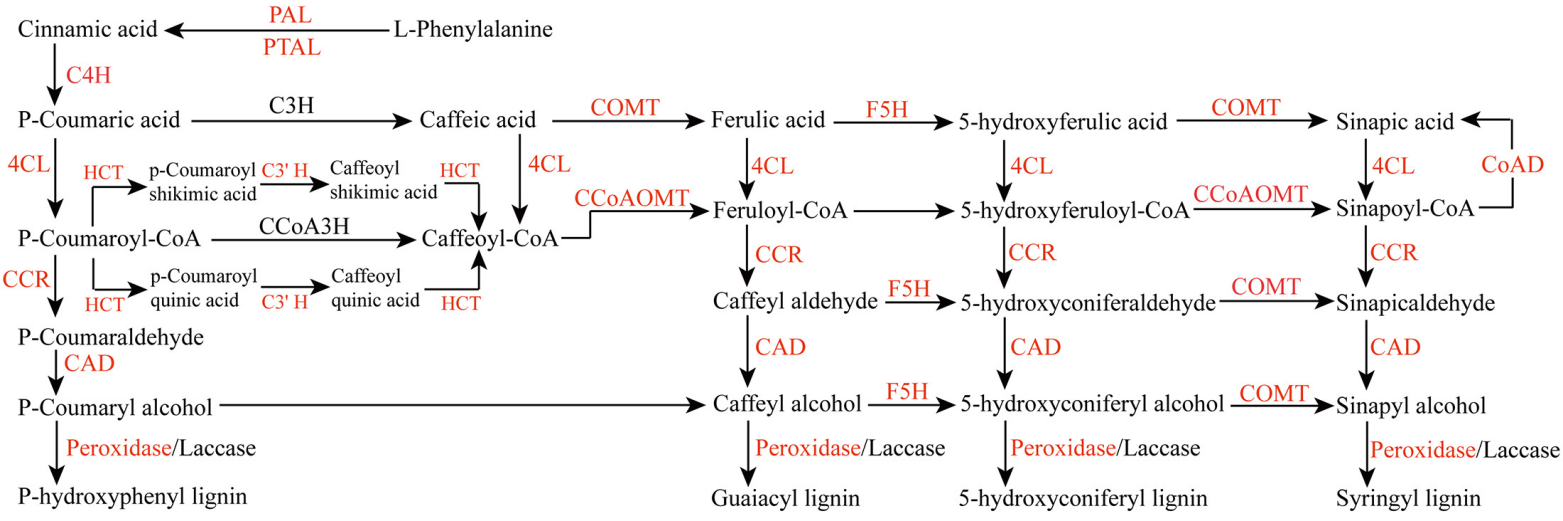
The diagram of metabolic pathway involved in lignin biosynthesis. It was completed according to Baucher et al. (1998) [71]. All metabolic enzymes predicted in *D*. *sinicus* transcriptome were marked with red font. 4CL: 4-coumarate CoA ligase; C3H: Coumarate 3-hdroxylase; C3′H: Coumaroylquinate (coumaroylshikimate) 3′-monooxygenase; C4H: Cinnamate 4-hydroxylase; CAD: Cinnamyl alcohol dehydrogenase; CCoAOMT: Caffeoyl-CoA 3-O-methyltransferase; CCR: Cinnamoyl-CoA reductase; COMT: Caffeic acid 3-O-methyltransferase; F5H: Ferulate 5-hydroxylase; HCT: Hydroxycinnamoyl-CoA: shikimate/quinate hydroxycinnamoyltransferase; PAL: Phenylalanine ammonia lyase; PTAL: Phenylalanine/tyrosine ammonia-lyase; PER: Peroxidase.

As is well known, the lignin content of bamboo is higher than most herbaceous plants [38], which may be due to the differences in the number or level of expression of key enzymes involved in lignin biosynthesis [18]. In the present study, 388 unigenes encoding 16 key enzymes, from the 81,744 unigenes, were identified involved in lignin biosynthesis (Table 5 and Fig 7). The numbers of putative unigenes related to lignin biosynthesis were compared based on Da bamboo of this study, Ma bamboo transcriptome data, Moso bamboo cDNA Database, and rice genes identified from the genome sequences, of which, genes encoding peroxidase remarkably had the most abundance. From the rice genome database [39], we found abundant of transcripts including 26 4-coumarate-CoA ligase (4CL), 23 laccase and 20 phenylalanine ammonia-lyase (PAL). These three genes are also abundant involved in lignin synthesis of Moso bamboo, Ma bamboo and Da bamboo. Particularly, some transcripts encoding Coumaroylquinate (coumaroylshikimate) 3'-monooxygenase (C3' H) and Shikimate O-hydroxycinnamoyltransferase (HCT) identified in Da bamboo, while they did not exist in other species. Similarly, 13 transcripts encoding Ferulate-5-hydroxylase (F5H) were found in Da bamboo, and only one of this gene was identified in Ma bamboo whereas it did not exist in Moso bamboo and rice. It would partially contribute to the increased Da bamboo lignin content in comparison to other species. There were transcripts encoding 5-hydroxyconiferyl aldehyde O-methyltransferase (AldOMT) in Moso bamboo and rice. However, AldOMT did not found in Da bamboo and Ma bamboo, which indicated that other genes encoding alternative methyltransferases, substituting for AldOMT activity, may exist in Da bamboo and Ma bamboo [18]. Also, 8 transcripts encoding Coniferyl-aldehyde dehydrogenase (CoAD) were found in Da bamboo and Ma bamboo, respectively. However, this transcript did not found in Moso bamboo. Da bamboo and Ma bamboo belong to *Dendrocalamus*, and Moso bamboo belongs to *Phyllostachys*. Thus, the difference of transcript number possibly represents lignin metabolism distinction in different genus. Among four species, the transcripts encoding Phenylalanine/tyrosine ammonia-lyase (PTAL) were found except rice, implying that PTAL might play an exclusive function in lignin biosynthesis of bamboo.

**Table 5:** Number of genes found in the bamboos and rice genome that encode key enzymes involved in the lignin biosynthesis pathway.

Enzymes	Da bamboo	Ma bamboo^a^	Moso Bamboo^b^	Rice^c^
4-coumarate-CoA ligase (4CL)	26	35	6	26
Caffeoyl caffeoyl-CoA O-methyltransferase (CCoAOMT)	9	4	8	10
Cinnamoyl-CoA reductase (CCR)	47	10	17	18
Caffeic acid O-methyltransferase (COMT)	2	7	2	10
Cinnamate-4-hydroxylase (C4H)	8	4	11	4
Cinnamoyl alcohol dehydrogenase (CAD)	15	2	5	21
Laccase	27	34	16	23
5-hydroxyconiferyl aldehyde O-methyltransferase (AldOMT)	0	0	2	7
3-deoxy-D-arabino-heptulosonate 7-phosphate synthase (DAHPS)	6	7	2	8
Coniferyl-aldehyde dehydrogenase (CoAD)	8	8	0	4
Coumaroylquinate (coumaroylshikimate) 3′-monooxygenase (C3′H)	6	0	0	0
Ferulate-5-hydroxylase (F5H)	13	1	3	0
Peroxidase	174	163	143	311
Phenylalanine ammonia-lyase (PAL)	15	17	13	20
Phenylalanine/tyrosine ammonia-lyase (PTAL)	5	1	14	0
Shikimate O-hydroxycinnamoyltransferase (HCT)	27	0	1	4

^a^The results were cited from Liu et al. (2012);

^b^The results were cited from Bamboo Genome Database (www.bamboogdb.org).

^c^The results were cited from Yu et al. (2002).

**Fig 8:**
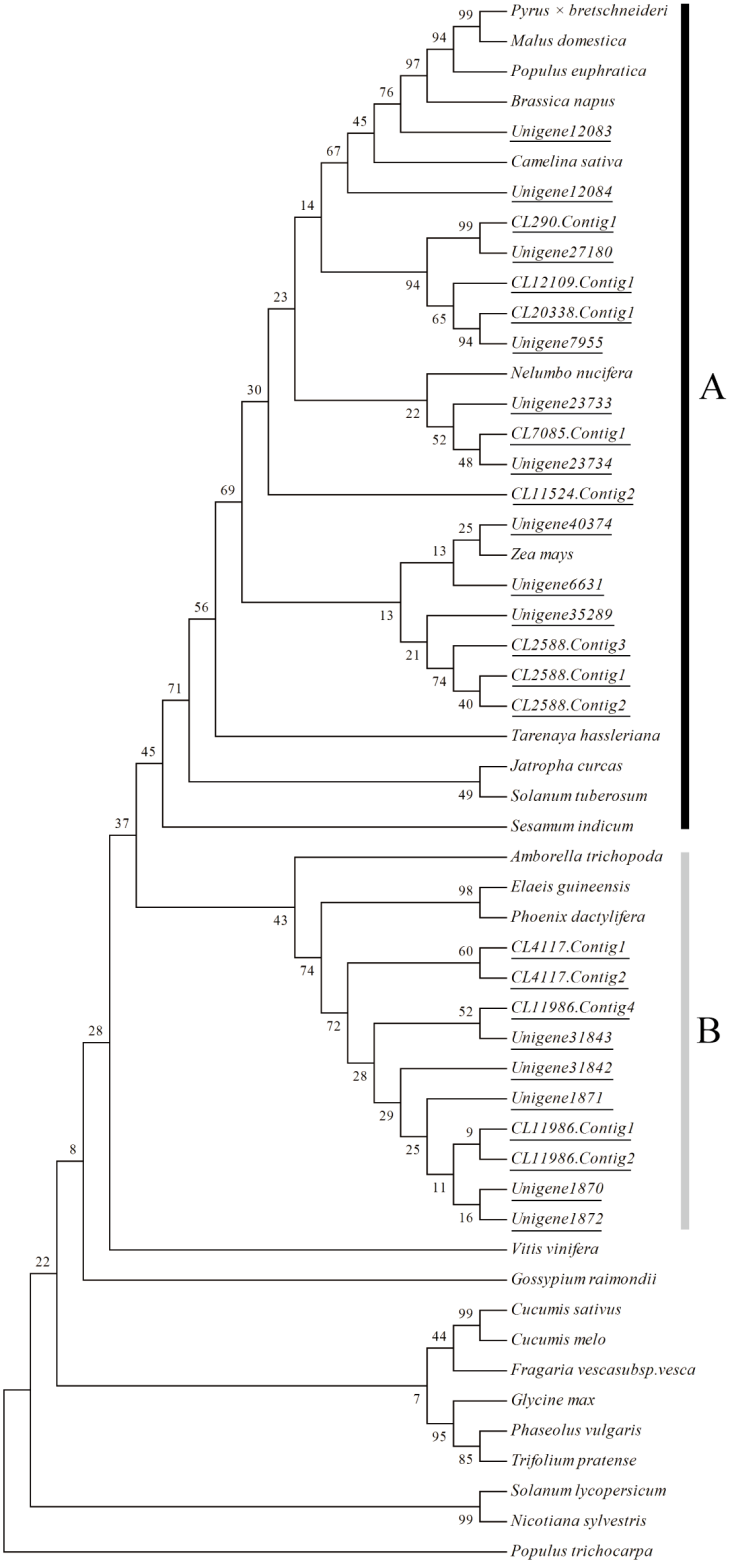
Phylogenic analysis of Shikimate O-hydroxycinnamoyltransferase. Two clades (A and B) that represent different likely enzymatic function of 27 *D*. *sinicus* unigenes were pointed. In the Fig, unigene number was denoted using underline. Besides *D*. *sinicus* transcriptome data, other protein sequences encoding Shikimate O-hydroxycinnamoyltransferase were obtained from NCBI. To distinctly display the relationship between *D*. *sinicus* and other species, the species name of each sequence was marked out, and the corresponding accession numbers were as follows: *Amborella trichopoda*, XP_011620549.1; *Brassica napus*, XP_013719902.1; *Camelina sativa*, XP_010478899.1; *Cucumis melo*, XP_008466864.1; *C*. *sativus*, NP_001295843.1; *Glycine max*, XP_003543709.1; *Gossypium raimondii*, XP_012445417.1; *Elaeis guineensis*, XP_010942061.1; *Fragaria vescasubsp*.*vesca*, XP_011467012.1; *Jatropha curcas*, XP_012071524.1; *Malus domestica*, XP_008380698.1; *Nelumbo nucifera*, XP_010256176.1; *Nicotiana sylvestris*, XP_009789449.1; *Phaseolus vulgaris*, AGV54440.1; *Phoenix dactylifera*, XP_008790160.1; *Populus euphratica*, XP_011031277.1; *P*. *trichocarpa*, XP_006368492.1; *Pyrus × bretschneideri*, XP_009344995.1; *Sesamum indicum*, XP_011073311.1; *Solanum tuberosum*, XP_006343633.1; *S*. *lycopersicum*, XP_004235891.1; *Tarenaya hassleriana*, XP_010545447.1; *Trifolium pretense*, ACI16630.1; *Vitis vinifera*, XP_002268988.1; *Zea mays*, XP_008673748.1.

Given many transcripts encoding HCT exclusively presented in Da bamboo, we searched its domain using database, and found that HCT contain a condensation domain. Thus, a phylogenetic tree was constructed by alignments of the protein sequences containing condensation domain, which can examine the phylogenetic relationship between the condensation domain proteins in *D*. *sinicus* and other plants (Fig 8). Based on the phylogenetic tree, twenty-seven sequences identified from *D*. *sinicus* were divided into two clades (A and B). In clade A, seventeen sequences were mainly classed to dicotyledon, however, in clade B, ten sequences were generally grouped with monocotyledon including *Elaeis guineensis* and *Phoenix dactylifera*, implying the structural or functional diversity of HCT unigenes in *D*. *sinicus*.

Generally, the transcript number involved in lignin biosynthesis in Moso bamboo is smaller than that in Da bamboo and Ma bamboo. The significant difference among Da bamboo, Ma bamboo and Moso bamboo is not surprising because the 10,608 Moso FL-cDNAs actually represent only one third to one fourth of the estimated total of Moso bamboo genes. Also, the Moso FL-cDNA libraries were constructed from shoots, leaves and roots from germinating seeds which were not the most representative tissues for high lignin content, while Da bamboo and Ma bamboo materials used for the transcriptome sequencing covered as many tissues as possible including culms of different developing periods [18]. The above results mean that the unique features of gene expression involved in lignin biosynthesis of Da bamboo.

### Functional genes involved in transcription factor and photosynthesis

According to the previous research, many transcription factor families play key roles in plant growth, development and immunity [40,41,42]. After BLASTn analysis, many unigenes were putatively identified as transcription factors, including ERF, Myb, Zinc finger, WRKY, Homeobox, bZIP, bHLH, NAC, MADS, etc (Table 6). Remarkably, Zinc finger had the most abundance in Ma bamboo and Da bamboo, while ERF was the most abundant in Moso bamboo. In the Da bamboo, the order of the number of transcription factor was as follows: Zinc finger, bHLH, Myb, WRKY, Homeobox, NAC, ERF, MADS, bZIP and CBF/NF-Y/archaeal histone, which was completely different from that of Moso bamboo, and was slightly different when compared with Ma bamboo. Taking into account that Da bamboo and Ma bamboo belong to sympodial bamboo and Moso bamboo was classified as scattered bamboo, the distinctions of abundance and order of the number of transcription factor might reflect the species differences.

**Table 6:** The most abundant transcription factors found in *D*. *sinicus* transcriptome.

Category of domain	Number		
	Moso bamboo^a^	Ma bamboo^b^	Da bamboo
ERF	71	74	121
Myb	41	212	157
Zinc finger (Including C2H2, CCCH, GATA, PHD and LIM)	39	419	772
WRKY	37	102	157
Homeobox	33	134	156
bZIP	27	41	36
bHLH	22	126	206
NAC	22	97	145
CBF/NF-Y/archaeal histone	15	25	29
MADS	12	62	85

^a^The results were cited from Peng et al. (2010);

^b^The results were cited from Liu et al. (2012).

**Fig 9:**
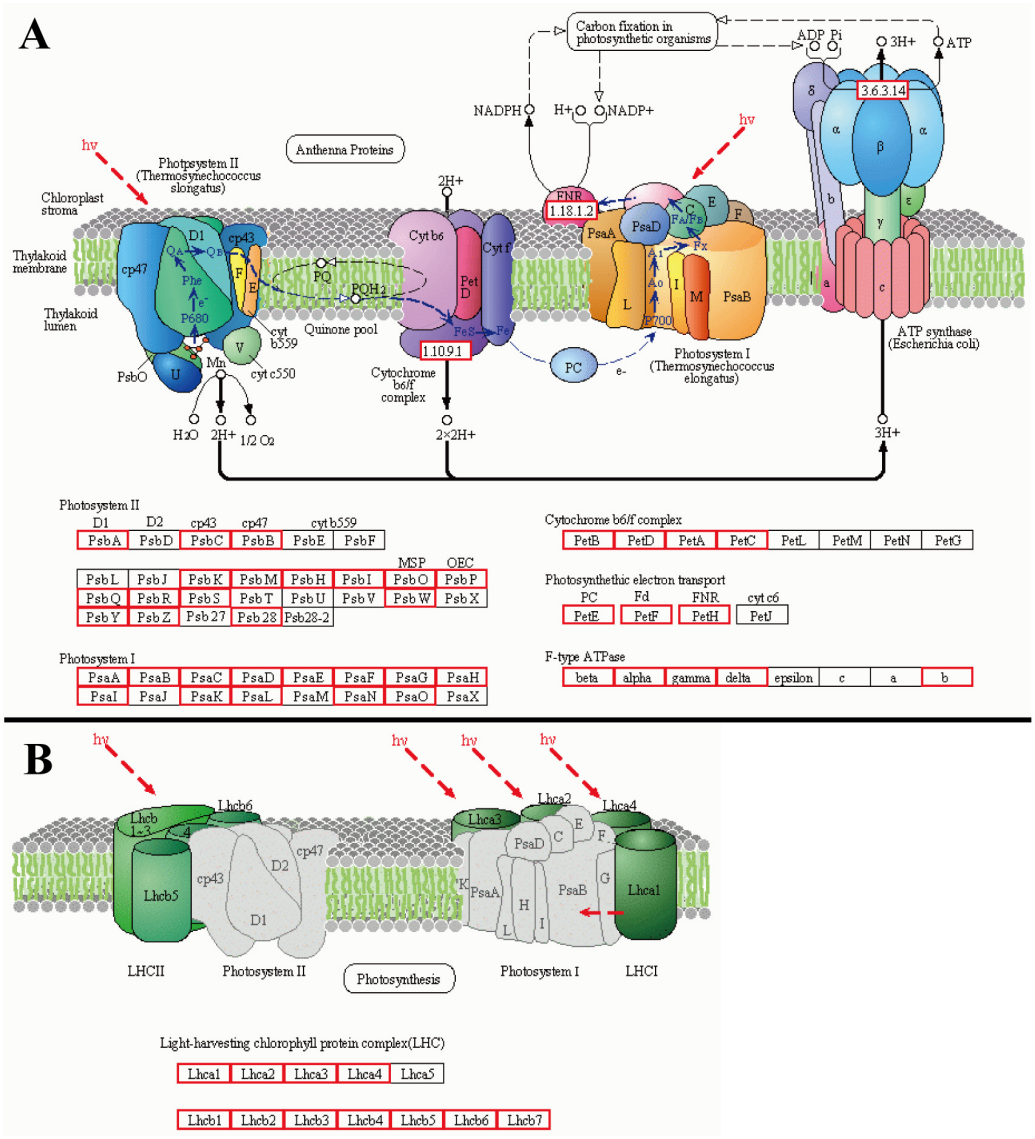
Unigenes of *D*. *sinicus* involved in photosynthesis (A) and antenna protein (B). The pathways are originated from KEGG. The enzymes detected in *D*. *sinicus* transcriptome were marked with red frame in the pathway. 1.18.1.2: Ferredoxin-NADP+ reductase; 1.10.9.1: cytochrome b6-f complex iron-sulfur subunit; 3.6.3.14: F-type H+-transporting ATPase subunit a; PsbA: photosystem II P680 reaction center D1 protein; PsbC: photosystem II CP43 chlorophyll apoprotein; PsbB: photosystem II CP47 chlorophyll apoprotein; PsbK: photosystem II PsbK protein; PsbM: photosystem II PsbM protein; PsbH: photosystem II PsbH protein; PsbI: photosystem II PsbI protein; PsbO: photosystem II oxygen-evolving enhancer protein 1; PsbP: photosystem II oxygen-evolving enhancer protein 2; PsbQ: photosystem II oxygen-evolving enhancer protein 3; PsbR: photosystem II 10kDa protein; PsbS: photosystem II 22kDa protein; PsbT: photosystem II PsbT protein; PsbW: photosystem II PsbW protein; PsbY: photosystem II PsbY protein; PsbZ: photosystem II PsbZ protein; Psb28: photosystem II 13kDa protein; PsaA: photosystem I P700 chlorophyll a apoprotein A1; PsaB: photosystem I P700 chlorophyll a apoprotein A2; PsaC: photosystem I subunit VII; PsaD: photosystem I subunit II; PsaE: photosystem I subunit IV; PsaF: photosystem I subunit III; PsaG: photosystem I subunit V; PsaH: photosystem I subunit VI; PsaI: photosystem I subunit VIII; PsaK: photosystem I subunit X; PsaL: photosystem I subunit XI; PsaN: photosystem I subunit PsaN; PsaO: photosystem I subunit PsaO; PetB: cytochrome b6; PetD: cytochrome b6-f complex subunit 4; PetA: apocytochrome f; PetC: cytochrome b6-f complex iron-sulfur subunit; PetE: plastocyanin; PetF: ferredoxin; PetH: ferredoxin—NADP+ reductase; beta: F-type H+-transporting ATPase subunit beta; alpha: F-type H+-transporting ATPase subunit alpha; gamma: F-type H+-transporting ATPase subunit gamma; delta: F-type H+-transporting ATPase subunit delta; b: F-type H+-transporting ATPase subunit b; Lhca: light-harvesting complex I chlorophyll a/b binding protein; Lhcb: light-harvesting complex II chlorophyll a/b binding protein.

**Fig 10:**
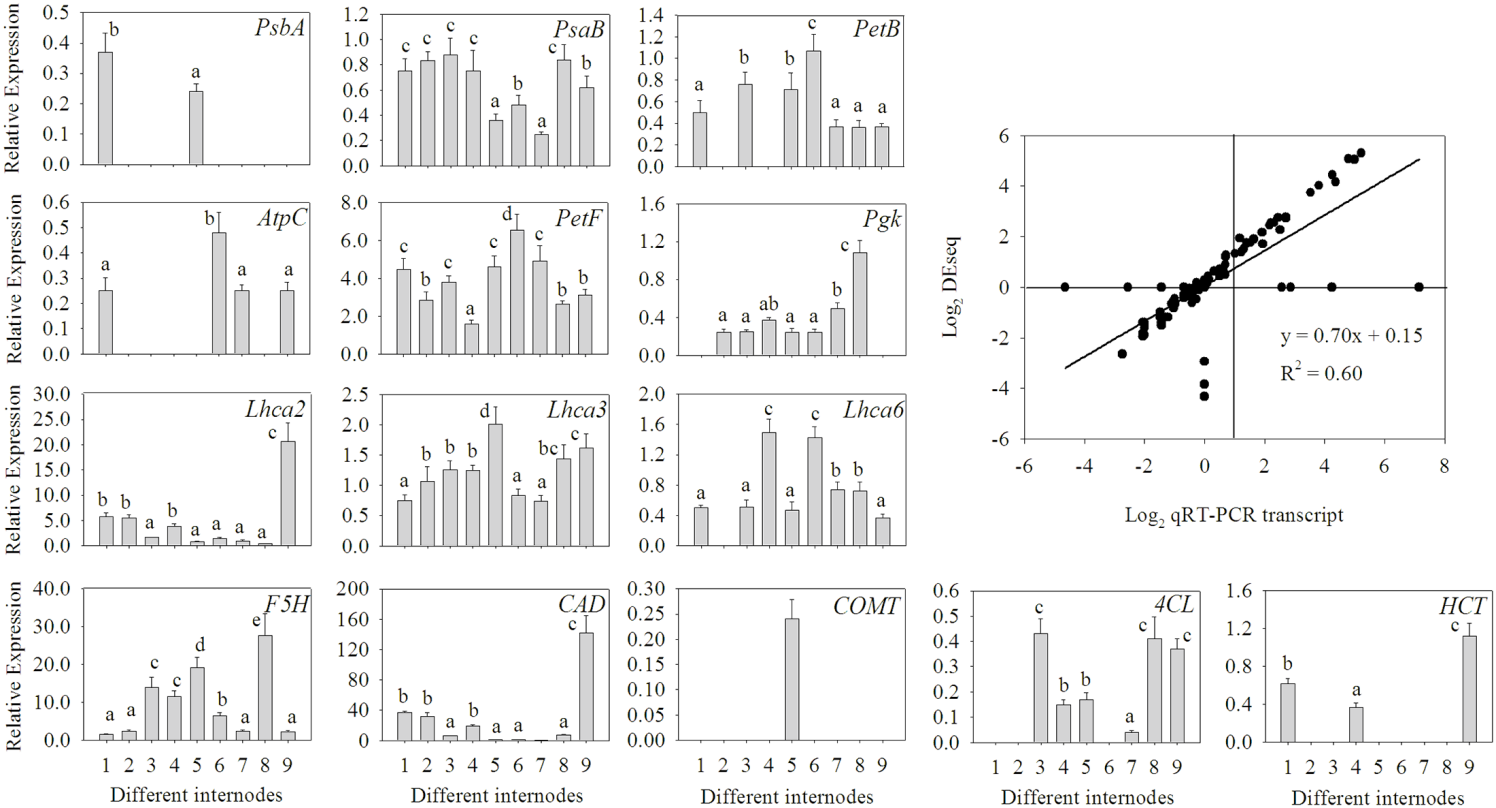
RT-qPCR validations of 14 genes involved in photosynthesis and lignin synthesis pathways. Different letters indicate differences at *P* ≤ 0.05 based on the LSD (least significant difference) test. Data are means of three replicates and each replicate was measured three times. The gene names and the primers used for RT-qPCR analysis are shown in Table 7. *AtpC*: F-type H^+^-transporting ATPase subunit c; *CAD*: Cinnamoyl alcohol dehydrogenase; *COMT*: Caffeic acid-3-O-methyltransferase; *HCT*: Shikimate O-hydroxycinnamoyltransferase; *PsbA*: photosystem II P680 reaction center D1 protein; *PsaB*: photosystem I P700 chlorophyll a apoprotein A2; *PetB*: cytochrome b6; *PetF*: ferredoxin; *Lhca2*: light-harvesting complex I chlorophyll a/b binding protein 2; *Lhca3*: light-harvesting complex I chlorophyll a/b binding protein 3; *Lhca6*: light-harvesting complex I chlorophyll a/b binding protein 6; *F5H*: ferulate-5-hydroxylase; *4CL*: 4-coumarate-CoA ligase.

A total of 109 transcripts encoding photosynthesis were identified (Fig 9A), including photosystem I and II, cytochrome b6/f complex, photosynthetic electron transport and F-type ATPase, and 24 transcripts were characterized as antenna proteins that regarded as the main tool for capturing light of plants (Fig 9B), which imply that culms involve in the photosynthesis during its height development, and that culm photosynthesis plays a certain role in the biomass production. In addition, to confirm the reliability of transcriptome data, the expression level of 14 unigenes, including *PsbA*, *PsaB*, *PetB* (chloroplast-encoded genes involved in photosynthesis), *PetF*, *Pgk*, *Lhca2*, *Lhca3*, *Lhca6* (nuclear-encoded genes involved in photosynthesis), *AtpC* (nuclear-encoded genes involved in Calvin cycle), and *F5H*, *CAD*, *COMT*, *4CL*, *HCT* (associated with lignin biosynthesis) were detected using real-time quantitative PCR (Fig 10). The results showed the expression level of 14 genes accorded with the transcriptome data.

**Table 7:** Quantitative PCR validation of the RNA-seq.

Gene ID	Description	Primer	
		Forward (5’- 3’)	Reverse (5’- 3’)
CL15529.Contig1	Photosystem II P680 reaction center D1 protein	GCTTGTTACATGGGTCGTGA	CTTCCTTGACCGATTGGGTA
CL12609.Contig1	Photosystem I P700 chlorophyll a apoprotein A2	GGGATTACAATCCGGAACAG	AAAGGCCCAAGGTATGGAAT
Unigene28575	Cytochrome b6	GGGTCGCAATGGCTTTATT	TCCACGGTCGAACTACCAG
CL8876.Contig4	Ferredoxin	GCCCTTGCCGTTTATTAGC	TGCCAAACAGCTTTTCGTT
Unigene21914	F-type H^+^-transporting ATPase subunit c	TCTTTCCTAAAAGCGGTGGA	CAATGCAATAGCTTCGGTGA
CL11294.Contig1	Light-harvesting complex I chlorophyll a/b binding protein 2	CCCCAAATGAGGTGTACGTT	CATGCATTGCTCCACAATTA
CL1328.Contig1	Light-harvesting complex II chlorophyll a/b binding protein 3	GGTCTTGGGTTTGCATTGTG	GGCGAAAGCATTCATGTTG
CL9897.Contig1	Light-harvesting complex II chlorophyll a/b binding protein 6	GACTCAGAGAAGCAGCAGCA	CATGCAAGCAATGAAGCAAC
CL756.Contig1	Phosphoglycerate kinase	ACATTTCAACGGGAGGTGGT	GTTGAAGAGGGGCAAACAAG
CL1818.Contig1	Ferulate-5-hydroxylase	TTAGTTCTCGGGCCGTTAAT	CACCCACAAGCAAAAATATCAC
CL4150.Contig1	Cinnamoyl alcohol dehydrogenase	CGAGGTAGTCAAGATGGATT	ACAGCTCACGAGCATGTACC
CL9744.Contig2	Caffeic acid-3-O-methyltransferase	TTCCTCTTGTTGCTGCTCCT	AGGGAGAAACCATGGCATTA
CL15267.Contig1	4-coumarate-CoA ligase	TCGCGACATCCAAACTATGA	GTCTAGGCACTGAAGCAACA
Unigene31843	Shikimate O-hydroxycinnamoyltransferase	TTCGACCGCACGGTAATCA	AGACTGACGATGCGCTGCTT

## Discussion

The significance of the BLAST comparison depends in part on the length of the query sequence, and short reads obtained from sequencing would rarely be matched to known genes [43]. In the present study, 78.71% of unigenes were annotated, which is relative high in contrast to other uncharacterized plant such as *Litsea cubeba* (56.00%) [37], *Siraitia grosvenorii* (59.90%) [26], *P*. *heterocycla* (69.75%) [19] and *D*. *latiflorus* (78.90%). The unmapped unigenes can be ascribed to the short sequence reads generated by the sequencing technology and the relatively short sequences of the resulting unigenes, most of which probably lack the conserved functional domains [44]. There are other possible reasons that some of these unigenes might be non-coding RNAs [18], and that the insufficient sequences of bamboo in public databases also influence the annotation efficiency [44]. Given the existence of species-specific genes, even among the species with high genomic synteny, many genes had big difference in their syntenic regions [45]. Thus, although the draft genome of moso bamboo [21], transcriptome data of other bamboo species [18,19] were reported, the present study is necessary to elucidate the clum development of *D*. *sinicus*.

Thiel et al. (2003) [46] indicated that approximately 3–7% of expressed genes contain putative SSR motifs, mainly within the un-translated regions of the mRNA. SSRs within gene sequences may have different putative functions, for example, SSR variations in 59-untranslated regions (UTRs) could regulate gene expression by affecting transcription and translation; SSR expansions in the 39-UTRs cause transcription slippage and produce expanded mRNA; intronic SSRs can affect gene transcription, mRNA splicing, or export to cytoplasm; SSRs within genes should be subjected to stronger selective pressure than other genomic regions [47]. In the present study, approximately 9% of the 7,353 unigenes contain single sequence repeat (SSR), which is similar to that of Ma bamboo (12.8%), and is much smaller than that of Moso bamboo (24%) and rice (44%). This result might be related to species difference and sampling strategy.

The synthesis of monolignols, lignin precursor, is implicated in many enzymatic reactions, which involves the general phenylpropanoid pathway starting with the deamination of phenylalanine and leading to the production of hydroxycinnamoyl CoA esters [48]. Although lignin is the most abundant phenylpropanoid derived from the hydroxycinnamoyl-CoA esters, the latter are also the precursors of a wide range of end products including flavonoids, anthocyanins and condensed tannins, which vary according to species, cell type and environmental signals [49]. In order to produce monolignols, hydroxycinnamoyl-CoA esters undergo successive hydroxylation and O-methylation of their aromatic rings involving the following enzymatic activities: shikimate *O*-hydroxycinnamoyltransferase (HCT); caffeoyl shikimate esterase (CSE); *p*-coumarate 3-hydroxylase (C3′H); caffeoyl CoA 3-*O*-methyltransferase (CCoAOMT); ferulate 5-hydroxylase (F5H) and caffeate/5-hydroxyferulate *O*-methyltransferase (COMT) [50,51].

HCT uses *p*-coumaroyl-CoA and caffeoyl-CoA as preferential substrates to transfer an acyl group to the acceptor compound shikimic acid, yielding *p*-coumaroyl shikimate. Usually, the differential expression of HCT gene was involved in defense against abiotic and biotic stress, for example, down regulation of HCT gene of *Leymus chinensis*'s leaves resulted from mechanical wounding [52], and down regulation of HCT gene was found when compared the susceptible with resisitant genotypes to leaf miner (*Leucoptera coffella*) [53]. Hoffmann et al. (2004) [54] pointed out that silencing of the HCT gene induced a dwarf phenotype and changes in lignin composition, suggesting that HCT catalyzes the reactions both immediately preceding and following the insertion of the 3-hydroxyl group by C3H into monolignol precursors. In this study, due to specific expression of HCT compared to other bamboos and rice, we conjecture that it might involve in the high lignin content and giant culm in *D*. *sinicus*.

In comparison to most other eukaryotes, plant genomes contain a higher proportion of recently duplicated genes [55]. These duplicates are mostly derived from segmental, whole genome duplication, and tandem duplication events [56,57]. Except segmental duplication and whole genome duplication, tandem duplication, which produces duplicates that are located in close proximity, has contributed significantly to the expansion of plant gene families [58,59]. Compared with the whole genome duplication, tandem duplications have more frequent occurrence and are responsible for much of the gene copy number and allelic variation within a population [60,61]. Although each tandem duplication event only affects a small number of genes, tandemly duplicated genes constitute approximately 14% of all duplicates in Arabidopsis [58]. In particular, genes involved in stress responses have an elevated probability of retention in a single-lineage fashion following tandem duplication, suggesting that these tandem duplicates are likely important for adaptive evolution to rapidly changing environments [62]. For example, in the *Eucalyptus grandis*, of the tandem duplicated genes *EgrHCT1-5*, only *EgrHCT4* and 5 are likely to be associated with developmental lignification; the other three have only residual expression in xylem and are inducible by abiotic stresses to different levels, suggesting neo- or subfunctionalization following duplication from a common ancestor [62]. In the present study, HCT gene families were divided into obviously different and regular clades, which is likely consistent with gene tandem duplication. This needs further study by quantitative analysis of gene expression.

Like as arbor species with stem, bamboos have culm that originated from the mother rhizome. The culm is covered by numerous overlapping sheaths that will shed off with the culm development. The growth process of culm is very strange. It initially hid under the ground surface, however, after growth starting, reached full height within a single growth season at an amazing elongation rate, *e*.*g*. for some bamboos, of over 100 cm per day [63]. It was also observed that culm did not show any diameter increment during or after the elongation period. The diameter with which it emerges remains unchanged throughout its life [25]. Because it is leafless and enwrapped by the rigid and nontransparent sheath during the culm development, the action of reserve nutrients was considered as the main source for the growth of bamboo culm and rhizome [25]. However, the transcripts encoding photosynthesis identified from culms indicated that culms photosynthesis associated with its height development.

Woody tissue photosynthesis has been hypothesized to have enabled the maintenance of biodiversity and terrestrial plant productivity throughout the Pleistocene, when atmospheric CO_2_ concentrations were only *c*. 200 ppm [64]. Even today, with atmospheric CO_2_ concentrations *c*. 400 ppm, recycling of CO_2_ by woody tissue photosynthesis can instantaneously offset between 7% and 98% of carbon loss in stems and branches of different species in summer, with a median of 72% [65,66]. The contribution of the woody tissue photosynthesis to the overall tree carbon balance largely differs between and even within tree species, depending on size, age and environment [67]. Irradiance is considered to be one of the major limiting factors because photosynthesis requires adequate photosynthetically active radiation to pass through the epidermal, peridermal, and/or rhytidomal layers to reach the light harvesting complexes of the chloroplasts [65,66,68]. In general, the productive months of culm emergence have days of longer photoperiod. Thus, it appears that the period of culm emergence also depend on climatic condition of locality [25]. Heavy shade of upper tree canopy inhibits bamboo regeneration and growth. It has been observed that clumps growing in the open sites produce culms of much better quality and quantity than the clumps growing under heavy shade [69]. Given that the leafless culm enwrapped by sheath and the culm usually grown under crowns, it seems that culms hardly capture enough light for assimilating carbon. According to views reported by Ávila *et al*. (2014) [65], the stem photosynthesis can be distinguished to two types: stem net photosynthesis (SNP), which includes net CO_2_ fixation by stems with stomata in the epidermis and net corticular CO_2_ fixation in suberized stems, and stem recycling photosynthesis (SRP), which defines CO_2_ recycling in suberized stems. It is widely accepted that the respiration of stem inner tissues is the CO_2_ source for SRP; however, labeled carbon transported upward by a xylem sap from roots to leaves is fixed in branches of *Platanus occidentalis* [70] and in detached branches of *Populus deltoids* [71], suggesting strongly that CO_2_ recycled by SNP or SRP may come also from the roots. Because development, maturation, and aging gradually performed from basal to top internodes, the expression pattern of functional gene among internodes diversified, which involved in up-regulated type, down-regulated type, fluctuant type, and discontinuous type. By real-time quantitative PCR analysis (Fig 10), discontinuous expression were displayed in some unigenes, including PsbA (chloroplast-encoded gene related to photosynthesis), AtpC (nuclear-encoded genes involved in Calvin cycle), and CAD, COMT, HCT (associated with lignin biosynthesis), indicating that the comlex expression pattern of genes resulted in development of *D*. *sinicus*. As has been noted, both SNP and SRP might involve in bamboo development. What’s more, the contribution rate of stem photosynthesis accounting for culm development and the carbon source of stem photosynthesis await further investigation.

## Supporting Information

S1 Table List of 128 KEGG pathways for unigenes. (XLSX)

## Abbreviations

COG: Clusters of orthologous groups
EST: Expression sequence tag
GO: Gene ontology
KEGG: Kyoto encyclopedia of genes and genomes
RNA-seq: RNA sequencing
FPKM: Fragments per kilobase of transcript per million fragments mapped
SRA: Sequence Read Archive
SSR: Simple sequence repeat

## References

[1] Meier MA (2011) Renewable Resources for Polymer Chemistry: A Sustainable Alternative? Macromol Rapid Comm 32: 1297–1298. 10.1002/marc.201100488 21837812

[2] Zhang X, Tu M, Paice MG (2011) Routes to potential bioproducts from lignocellulosic biomass lignin and hemicelluloses. BioEnergy Research 4: 246–257.

[3] FitzPatrick M, Champagne P, Cunningham MF, Whitney RA (2010) A biorefinery processing perspective: treatment of lignocellulosic materials for the production of value-added products. Bioresource Technol 101: 8915–8922. 10.1016/j.biortech.2010.06.125 20667714

[4] Hansen NM, Plackett D (2008) Sustainable films and coatings from hemicelluloses: a review. Biomacromolecules 9: 1493–1505. 10.1021/bm800053z 18457452

[5] Ohrnberger D (1999) The bamboos of the world: annotated nomenclature and literature of the species and the higher and lower taxa. Elsevier Science, Amsterdam, p. 12–38.

[6] Hui CM, Yang XY, Liang N, Chen F (2014) A study on the conservation and development of *Dendrocalamus sinicus* form Yunnan, China. Appl Mech Mater 522–524: 1084–1088.

[7] Yi T, Shi J, Ma L, Wang H, Yang L (2008) Iconographia Bambusoidearum Sinicarum. Science Press, Beijing, China.

[8] Shi ZJ, Xiao LP, Deng J, Sun RC (2013) Isolation and structural characterization of lignin polymer from *Dendrocalamus sinicus*. Bioenerg Res 6: 1212–1222.

[9] Cui K, He C, Zhang JG, Duan AG, Zeng YF (2012) Temporal and spatial profiling of Internode elongation-associated protein expression in rapidly growing culms of bamboo. J Proteome Res 11: 2492–2507. 10.1021/pr2011878 22397471

[10] Chen CY, Hsieh MH, Yang CC, Lin CS, Wang AY (2010) Analysis of the cellulose synthase genes associated with primary cell wall synthesis in *Bambusa oldhamii*. Phytochemistry 71: 1270–1279. 10.1016/j.phytochem.2010.05.011 20541781

[11] Peng ZH, Lu TH, Li LB, Liu XH, Gao ZM, et al (2010) Genome-wide characterization of the biggest grass, bamboo, based on 10, 608 putative full-length cDNA sequences. BMC Plant Biol 10: 116 10.1186/1471-2229-10-116 20565830 PMC3017805

[12] Chen TH, Huang YC, Yang CS, Yang CC, Wang AY, et al (2009) Insights into the catalytic properties of bamboo vacuolar invertase through mutational analysis of active site residues. Phytochemistry 70: 25–31. 10.1016/j.phytochem.2008.10.004 19010503

[13] Chiu WB, Lin CH, Chang CJ, Hsieh MH, Wang AY (2006) Molecular characterization and expression of four cDNAs encoding sucrose synthase from green bamboo *Bambusa oldhamii*. New Phytol 170: 53–63. 16539603 10.1111/j.1469-8137.2005.01638.x

[14] Hsieh CW, Liu LK, Yeh SH, Chen CF, Lin HI, et al (2006) Molecular cloning and functional identification of invertase isozymes from green bamboo *Bambusa oldhamii*. J Agric Food Chem 54: 3101–3107. 16608237 10.1021/jf052711s

[15] Yang L, Lou Y, Peng Z, Zhao H, Sun H, et al (2015) Molecular characterization and primary functional analysis of PeMPEC, a magnesium-protoporphyrin IX monomethyl ester cyclase gene of bamboo (*Phyllostachys edulis*). Plant Cell Rep: 1–11. 10.1007/s00299-015-1846-1 26215310

[16] Jiang Z, Peng Z, Gao Z, Liu C, Yang C (2012) Characterization of different isoforms of the light-harvesting chlorophyll a/b complexes of photosystem II in bamboo. Photosynthetica 50: 129–138.

[17] Zhou MB, Yang P, Gao PJ, Tang DQ (2011) Identification of differentially expressed sequence tags in rapidly elongating *Phyllostachys pubescens* internodes by suppressive subtractive hybridization. Plant Mol Biol Rep 29: 224–231.

[18] Liu M, Qiao G, Jiang J, Yang H, Xie L, et al (2012) Transcriptome sequencing and de novo analysis for ma bamboo (*Dendrocalamus latiflorus* Munro) using the Illumina platform. PloS one 7: e46766 10.1371/journal.pone.0046766 23056442 PMC3463524

[19] He CY, Cui K, Zhang JG, Duan AG, Zeng YF (2013) Next-generation sequencing-based mRNA and microRNA expression profiling analysis revealed pathways involved in the rapid growth of developing culms in Moso bamboo. BMC Plant Biol: 119. 10.1186/1471-2229-13-119 PMC3765735 23964682

[20] Wu YJ, Chen HM, Wu TT, Wu JS, Chu RM, et al (2006) Preparation of monoclonal antibody bank against whole water-soluble proteins from rapid-growing bamboo shoots. Proteomics 6: 5898–5902. 17051642 10.1002/pmic.200600278

[21] Peng Z, Lu Y, Li L, Zhao Q, Feng Q, et al (2013) The draft genome of the fast-growing non-timber forest species moso bamboo (*Phyllostachys heterocycla*). Nat Genet 45: 456–461. 10.1038/ng.2569 23435089

[22] Dong YR, Zhang ZR, Yang HQ (2012) Sixteen novel microsatellite markers developed for *Dendrocalamus sinicus* (Poaceae), the strongest woody bamboo in the world. Am J Bot 99: e347–e349. 10.3732/ajb.1200029 22933358

[23] Wang Z, Gerstein M, Snyder M (2009) RNA-Seq: a revolutionary tool for transcriptomics. Nat Rev Genet 10: 57–63. 10.1038/nrg2484 19015660 PMC2949280

[24] Wang Z, Fang B, Chen J, Zhang X, Luo Z, et al (2010) De novo assembly and characterization of root transcriptome using Illumina paired-end sequencing and development of cSSR markers in sweetpotato (*Ipomoea batatas*). BMC genomics 11: 726 10.1186/1471-2164-11-726 21182800 PMC3016421

[25] Banik RL (2015) Morphology and growth In: Liese W and Köhl M, editor. Bamboo. Spinger International Publishing, Berlin, p. 43–90.

[26] Tang Q, Ma X, Mo C, Wilson IW, Song C, et al (2011) An efficient approach to finding Siraitia grosvenorii triterpene biosynthetic genes by RNA-seq and digital gene expression analysis. BMC genomics 12: 343 10.1186/1471-2164-12-343 21729270 PMC3161973

[27] Xie F, Burklew CE, Yang Y, Liu M, Xiao P, et al (2012) De novo sequencing and a comprehensive analysis of purple sweet potato (Impomoea batatas L.) transcriptome. Planta 236: 101–113. 10.1007/s00425-012-1591-4 22270559

[28] Grabherr MG, Haas BJ, Yassour M, Levin JZ, Thompson DA, et al (2011) Full-length transcriptome assembly from RNA-Seq data without a reference genome. Nat Biotechnol 29: 644–652. 10.1038/nbt.1883 21572440 PMC3571712

[29] Iseli C, Jongeneel CV, Bucher P (1999) ESTScan: a program for detecting, evaluating, and reconstructing potential coding regions in EST sequences. Proc Int Conf Intell Syst Mol Biol 99:: 138–148. 10786296

[30] Grabherr MG, Haas BJ, Yassour M, Levin JZ, Thompson DA, Amit I, Adiconis X, Fan L, Raychowdhury R, Zeng Q (2011) Full-length transcriptome assembly from RNA-Seq data without a reference genome. Nat Biotechnol 29: 644–652. 10.1038/nbt.1883 21572440 PMC3571712

[31] Conesa A, Götz S, García-Gómez JM, Terol J, Talón M, et al (2005) Blast2GO: a universal tool for annotation, visualization and analysis in functional genomics research. Bioinformatics 21: 3674–3676. 16081474 10.1093/bioinformatics/bti610

[32] Ye J, Fang L, Zheng H, Zhang Y, Chen J, et al (2006) WEGO: a web tool for plotting GO annotations. Nucleic Acids Res 34: W293–W297. 16845012 10.1093/nar/gkl031 PMC1538768

[33] Wei W, Qi X, Wang L, Zhang Y, Hua W, et al (2011) Characterization of the sesame (*Sesamum indicum* L.) global transcriptome using Illumina paired-end sequencing and development of EST-SSR markers. BMC genomics 12: 451 10.1186/1471-2164-12-451 21929789 PMC3184296

[34] Zeng S, Xiao G, Guo J, Fei Z, Xu Y, et al (2010) Development of a EST dataset and characterization of EST-SSRs in a traditional Chinese medicinal plant, *Epimedium sagittatum* (Sieb. Et Zucc.) Maxim. BMC genomics 11: 94 10.1186/1471-2164-11-94 20141623 PMC2829513

[35] Li R, Li Y, Fang X, Yang H, Wang J, et al (2009) SNP detection for massively parallel whole-genome resequencing. Genome Res 19: 1124–1132. 10.1101/gr.088013.108 19420381 PMC2694485

[36] Li R, Zhu H, Ruan J, Qian W, Fang X, et al (2010) De novo assembly of human genomes with massively parallel short read sequencing. Genome Res 20: 265–272. 10.1101/gr.097261.109 20019144 PMC2813482

[37] Han XJ, Wang YD, Chen YC, Lin LY, Wu QK (2013) Transcriptome sequencing and expression analysis of terpenoid biosynthesis genes in *Litsea cubeba*. PloS one 8: e76890 10.1371/journal.pone.0076890 24130803 PMC3793921

[38] Scurlock J, Dayton D, Hames B (2000) Bamboo: an overlooked biomass resource? Biomass Bioenerg 19: 229–244.

[39] Yu J, Hu S, Wang J, Wong GK, Li S, et al (2002) A draft sequence of the rice genome (*Oryza sativa* L. ssp. *indica*). Science 296: 79–92. 11935017 10.1126/science.1068037

[40] Ariizumi T, Lawrence PK, Steber CM (2011) The role of two F-box proteins, SLEEPY1 and SNEEZY, in *Arabidopsis* gibberellin signaling. Plant Physiol 155: 765–775. 10.1104/pp.110.166272 21163960 PMC3032465

[41] Ling J, Jiang W, Zhang Y, Yu H, Mao Z, et al (2011) Genome-wide analysis of WRKY gene family in *Cucumis sativus*. BMC genomics 12: 471 10.1186/1471-2164-12-471 21955985 PMC3191544

[42] Reyes JC, Muro-Pastor MI, Florencio FJ (2004) The GATA family of transcription factors in *Arabidopsis* and rice. Plant Physiol 134: 1718–1732. 15084732 10.1104/pp.103.037788 PMC419845

[43] Novaes E, Drost DR, Farmerie WG, Pappas GJ, Grattapaglia D, et al (2008) High-throughput gene and SNP discovery in *Eucalyptus grandis*, an uncharacterized genome. BMC genomics 9: 312 10.1186/1471-2164-9-312 18590545 PMC2483731

[44] Hou R, Bao Z, Wang S, Su H, Li Y, et al (2011) Transcriptome sequencing and de novo analysis for Yesso scallop (*Patinopecten yessoensis*) using 454 GS FLX. PloS one 6: e21560 10.1371/journal.pone.0021560 21720557 PMC3123371

[45] Gui YJ, Zhou Y, Wang Y, Wang S, Wang SY, et al (2010) Insights into the bamboo genome: syntenic relationships to rice and sorghum. J Integra Plant Biol 52: 1008–1015. 10.1111/j.1744-7909.2010.00965.x 20977658

[46] Thiel T, Michalek W, Varshney R, Graner A (2003) Exploiting EST databases for the development and characterization of gene-derived SSR-markers in barley (*Hordeum vulgare* L.). Theor Appl Genet 106: 411–422. 12589540 10.1007/s00122-002-1031-0

[47] Li YC, Korol AB, Fahima T, Nevo E (2004) Microsatellites within genes: structure, function, and evolution. Mol Biol Evol 21: 991–1007. 14963101 10.1093/molbev/msh073

[48] Carocha V, Soler M, Hefer C, Cassan-Wang H, Fevereiro P, et al (2015) Genome-wide analysis of the lignin toolbox of *Eucalyptus grandis*. New Phytol 206: 1297–1313. 10.1111/nph.13313 25684249

[49] Dixon RA, Paiva NL (1995) Stress-induced phenylpropanoid metabolism. Plant Cell 7: 1085 12242399 10.1105/tpc.7.7.1085 PMC160915

[50] Boerjan W, Ralph J, Baucher M (2003) Lignin biosynthesis. Annu Rev plant biol 54: 519–546. 14503002 10.1146/annurev.arplant.54.031902.134938

[51] Vanholme R, Cesarino I, Rataj K, Xiao Y, Sundin L, et al (2013) Caffeoyl shikimate esterase (CSE) is an enzyme in the lignin biosynthetic pathway in *Arabidopsis*. Science 341: 1103–1106. 10.1126/science.1241602 23950498

[52] Chen S, Cai Y, Zhang L, Yan X, Cheng L, et al (2014) Transcriptome analysis reveals common and distinct mechanisms for sheepgrass (*Leymus chinensis*) responses to defoliation compared to mechanical wounding. PloS one 9: e89495 10.1371/journal.pone.0089495 24586824 PMC3931765

[53] Cardoso DC, Martinati JC, Giachetto PF, Vidal RO, Carazzolle MF, et al (2014) Large-scale analysis of differential gene expression in coffee genotypes resistant and susceptible to leaf miner–toward the identification of candidate genes for marker assisted-selection. BMC genomics 15: 66 10.1186/1471-2164-15-66 24460833 PMC3924705

[54] Hoffmann L, Besseau S, Geoffroy P, Ritzenthaler C, Meyer D, et al (2004) Silencing of hydroxycinnamoyl-coenzyme a shikimate/quinate hydroxycinnamoyltransferase affects phenylpropanoid biosynthesis. Plant Cell 16: 1446–1465. 15161961 10.1105/tpc.020297 PMC490038

[55] Lockton S, Gaut BS (2005) Plant conserved non-coding sequences and paralogue evolution. Trends Genet 21: 60–65. 15680516 10.1016/j.tig.2004.11.013

[56] Initiative AG (2000) Analysis of the genome sequence of the flowering plant *Arabidopsis thaliana*. Nature 408: 796 11130711 10.1038/35048692

[57] Tuskan GA, Difazio S, Jansson S, Bohlmann J, Grigoriev I, et al (2006) The genome of black cottonwood, *Populus trichocarpa* (Torr. & Gray). Science 313: 1596–1604. 16973872 10.1126/science.1128691

[58] Rizzon C, Ponger L, Gaut BS (2006) Striking similarities in the genomic distribution of tandemly arrayed genes in Arabidopsis and rice. PLoS Comput Biol 2: e115 16948529 10.1371/journal.pcbi.0020115 PMC1557586

[59] Zhang L, Gaut BS (2003) Does recombination shape the distribution and evolution of tandemly arrayed genes (TAGs) in the *Arabidopsis thaliana* genome? Genome Res 13: 2533–2540. 14656961 10.1101/gr.1318503 PMC403795

[60] Clark RM, Schweikert G, Toomajian C, Ossowski S, Zeller G, et al (2007) Common sequence polymorphisms shaping genetic diversity in *Arabidopsis thaliana*. Science 317: 338–342. 17641193 10.1126/science.1138632

[61] Rostoks N, Borevitz JO, Hedley PE, Russell J, Mudie S, et al (2005) Single-feature polymorphism discovery in the barley transcriptome. Genome biol 6: R54 15960806 10.1186/gb-2005-6-6-r54 PMC1175974

[62] Hanada K, Zou C, Lehti-Shiu MD, Shinozaki K, Shiu SH (2008) Importance of lineage-specific expansion of plant tandem duplicates in the adaptive response to environmental stimuli. Plant Physiol 148: 993–1003. 10.1104/pp.108.122457 18715958 PMC2556807

[63] Ueda K (1960) Studies on the physiology of bamboo with reference to its practical application. Bull Kyoto Univ Forests 30: 1–167.

[64] Busch FA, Sage TL, Cousins AB, Sage RF (2013) C3 plants enhance rates of photosynthesis by reassimilating photorespired and respired CO_2_. Plant Cell Environ 36: 200–212. 10.1111/j.1365-3040.2012.02567.x 22734462

[65] Ávila E, Herrera A, Tezara W (2014) Contribution of stem CO_2_ fixation to whole-plant carbon balance in nonsucculent species. Photosynthetica 52: 3–15.

[66] Teskey RO, Saveyn A, Steppe K, McGuire MA (2008) Origin, fate and significance of CO_2_ in tree stems. New Phytol 177: 17–32. 18028298 10.1111/j.1469-8137.2007.02286.x

[67] Vandegehuchte MW, Bloemen J, Vergeynst LL, Steppe K (2015) Woody tissue photosynthesis in trees: salve on the wounds of drought? New Phytol: 1–5. 10.1111/nph.13599 26226885

[68] Sun Q, Yoda K, Suzuki M, Suzuki H (2003) Vascular tissue in the stem and roots of woody plants can conduct light. J Exp Bot 54: 1627–1635. 12730266 10.1093/jxb/erg167

[69] Banik R (2000) Silviculture and field-guide to priority bamboos of Bangladesh and South Asia. BFRI, Chittagong, p. 1–187.

[70] McGuire M, Marshall J, Teskey R (2009) Assimilation of xylem-transported ^13^C-labelled CO_2_ in leaves and branches of sycamore (*Platanus occidentalis* L.). J Exp Bot 60: 3809–3817. 10.1093/jxb/erp222 19602545 PMC2736895

[71] Bloemen J, McGuire MA, Aubrey DP, Teskey RO, Steppe K (2013) Assimilation of xylem-transported CO_2_ is dependent on transpiration rate but is small relative to atmospheric fixation. J Exp Bot 64: 2129–2138. 10.1093/jxb/ert071 23580747

